# Is Eating Raisins Healthy?

**DOI:** 10.3390/nu12010054

**Published:** 2019-12-24

**Authors:** Alexandra Olmo-Cunillera, Danilo Escobar-Avello, Andy J. Pérez, María Marhuenda-Muñoz, Rosa Mª Lamuela-Raventós, Anna Vallverdú-Queralt

**Affiliations:** 1 Department of Nutrition, Food Science and Gastronomy, School of Pharmacy and Food Sciences and XaRTA, Institute of Nutrition and Food Safety (INSA-UB), University of Barcelona, 08921 Santa Coloma de Gramenet, Spain; alexandra.olmo@ub.edu (A.O.-C.); daniescobar01@gmail.com (D.E.-A.); mmarhuendam@ub.edu (M.M.-M.); lamuela@ub.edu (R.M.L.-R.); 2 Departmento de Análisis Instrumental, Facultad de Farmacia, Universidad de Concepción, Concepción 4191996, Chile; aperezd@udec.cl; 3 Consorcio CIBER, M.P. Fisiopatología de la Obesidad y Nutrición (CIBERObn), Instituto de Salud Carlos III (ISCIII), 28029 Madrid, Spain

**Keywords:** dried fruits, polyphenols, tartaric acid, prebiotics, fiber, glycemic index, cardiovascular health, diabetes, antioxidant capacity, dental health

## Abstract

Raisins are dried grapes consumed worldwide that contain beneficial components for human health. They are rich in fiber and phytochemicals such as phenolic compounds. Despite a 60% sugar content, several studies have reported health-promoting properties for raisins and this review compiles the intervention studies, as well as the cell line and animal model studies carried out to date. It has been demonstrated that raisins possess a low-to-moderate glycemic index, which makes them a healthy snack. They seem to contribute to a better diet quality and may reduce appetite. Their antioxidant capacity has been correlated to the phenolic content and this may be involved in the improvement of cardiovascular health. In addition, raisins maintain a good oral health due to their antibacterial activity, low adherence to teeth and an optimum oral pH. Raisin consumption also seems to be favorable for colon function, although more studies should be done to conclude this benefit. Moreover, gut microbiota could be affected by the prebiotic content of raisins. Cell line and animal model studies show other potential benefits in specific diseases, such as cancer and Alzheimer’s disease. However, deeper research is required and future intervention studies with humans are needed. Overall, incorporating an 80–90 g portion of raisins (half a cup) into the daily diet may be favorable for human health.

## 1. Introduction

Raisins are dried grapes mostly obtained from different cultivars of *Vitis vinifera* L. and are extensively consumed worldwide. The type of raisin depends on the grape variety, color and size. The most common are dark raisins, usually obtained from Thompson seedless grapes. Golden raisins, or Muscats, are normally produced from white Muscat grapes. Sultanas originate from seedless yellow grapes and are usually sweeter and softer than other varieties. Zante currants, currants or Corinthian raisins are produced from black Corinth grapes and are smaller in size [1].

Raisins are sweet as they consist of about 60% sugar, predominantly fructose and glucose [2], which gives rise to the common conception that they are unhealthy. However, they are rich in dietary fiber (3.3–4.5 g per 100 g) (Table 1) [2,3], which contributes to their prebiotic effect [4,5], as they are selectively used by host microorganisms and confer a health benefit [6]. During the production of raisins, the dehydration process converts part of the grape sugars into fructan, a form of fiber. While fructans are not detectable in grapes, in raisins, the fructan content can be up to 8% [3]. In plants, fructans are synthesized from sucrose by the action of two or more different fructosyltransferases [7].

Furthermore, raisins represent an important source of potassium and other bioactive compounds, including phenolic compounds and tartaric acid, which may benefit human health [1]. The growing interest in phytochemicals lies in their biological and physiological activities with health-promoting attributes. Polyphenols are plant secondary metabolites and are reported to have multiple biological effects [9,10]. The major polyphenols found in raisins are phenolic acids (caftaric and coutaric acid) and flavonols (quercetin and kaempferol glycosides, and rutin) [11,12,13]. Anthocyanins have also been identified [14]. Both the total and individual phenolic content vary widely among different raisin varieties [14,15]. Other minor phytochemicals found in raisins are triterpenoids (oleanolic acid, oleanolic aldehyde, betulin and betulinic acid) [16] and tartaric acid, which works synergistically with fiber to maintain a healthy digestive system [17].

Despite having these beneficial components, some authors have also described the presence of ochratoxin A (OTA) in raisins. OTA is a mycotoxin produced by *Aspergillus ochraceus* and other *Aspergillus* species, to which carcinogenic, nephrotoxic, teratogenic, immunotoxic and possible neurotoxic properties have been attributed [18,19]. Raisins can become contaminated with the fungus if there is a spell of humid weather during the drying process [20]. Consequently, the European Commission has established a maximum level of 10 µg/kg for OTA in dried vine fruit [21]. Although several studies have found mycotoxin levels in raisins to be below the safety limit [18,22,23], others have reported samples that exceed it [19]. Ostry et al. [20] estimated the dietary exposure dose of OTA from raisins for children and adults and found that the risk of an acute toxic effect was minimal. Although there might be a risk of delayed toxic effects (particularly carcinogenic) after the ingestion of very low single or repeated doses of OTA, this may be outweighed by the health-promoting properties of raisins.

Thus, the aim of this review is to compile the intervention studies carried out so far on raisins and their beneficial impact on human health. To do so, the words “raisin or raisins” and “health benefits” were used for searching on Scopus and Pubmed. Moreover, the cell line and animal model studies resulting from the search have also been incorporated. Despite not being considered as prestigious as human studies, they can also reveal knowledge about molecular action mechanisms and prove an approximation for humans.

## 2. Antioxidant Capacity

Reactive oxygen species (ROS) are known to contribute to various degenerative diseases [24]. Antioxidants have a great potential to prevent and diminish the formation of these species. Therefore, eating food rich in antioxidants may help reduce the risk of suffering from these diseases.

Raisins show excellent antioxidant capacity in both in vitro and in vivo experiments. The antibacterial and antioxidant activity of raisins comes mainly from their content of phenolic compounds. Numerous studies have shown a positive correlation between the antioxidant capacity and total phenol content in raisins [12,25,26,27,28,29]. Specific polyphenols such as catechin, procyanidins, and quercetin have been correlated with antioxidant and antimicrobial activities [12,30].

Raisins were found to be the solid food, within the ones studied, with the highest content of total phenols (mg/kg) (raisins > tarhana > dried black plum > dried apricot > red grape > fresh paprika > grape > paprika paste > fresh black plum > *Urtica* sp. > cherry > paprika pickle > fresh apricot). Further, a correlation between total phenols and total antioxidant activity was demonstrated [26].

The levels of total phenolic compounds differed between varieties of raisins, being white raisins the ones with the lowest value and red raisins the ones with the highest value [27]. This was in accordance with the antioxidant capacity: white raisins had the lowest value, whereas red raisins had the highest. A correlation between total phenolic content and antioxidant capacity was demonstrated again.

When analyzing phenolic groups of raisins separately, a correlation between total phenolic acids and antioxidant activity was found, being ferulic acid the one with the highest correlation, followed by *trans*-caftaric and *trans*-coutaric acid. In the case of total flavonol, there was also a positive correlation, being myricetin-3-*O*-glucoside the one with the highest correlation, followed by rutin, kaempferol-3-*O*-glucoside and quercetin-3-*O*-glucoside [15].

Although total antioxidant capacity assays in plasma have many detractors [31], few reports have studied the antioxidant capacity of raisins in plasma. Kanellos et al. [32] demonstrated that the consumption of 144 g of Corinthian raisins by healthy individuals was related to an increased resistance of serum to oxidation in the postprandial state, which in turn was associated with an increase of phenolic compounds in serum plasma. However, whether the increase in antioxidant capacity is a direct effect of the phenolic compounds could not be concluded, since other antioxidants could contribute. The peak of plasma total phenolic content and serum oxidation resistance showed up 1 h after raisin consumption.

In a crossover study, Parker et al. [11] compared the serum antioxidant capacity in 15 healthy human males with the phenolic content of green Thompson seedless grapes, sun-dried raisins and golden raisins over four weeks. Volunteers consumed 250 g of fresh Thompson seedless grapes, 50 g of sun-dried raisins, or 50 g of golden raisins every day. Serum and plasma samples were collected 1 and 2 h after consumption once a week. Long-term consumption of raisins/grapes increased serum antioxidant capacity by the second and third week, although values dropped in the fourth week. The authors speculated that there might be a physiological plateau after two or three weeks of continuous ingestion. A positive correlation between increased phenolic content and increased antioxidant capacity in vitro was reported. It seems that there are positive benefits to eating raisins each day over a long period.

Although it is unquestionable that raisins possess a great antioxidant capacity and phenolic compounds may play a crucial role, this kind of studies are not enough to prove the capacity of raisins to decrease risk of some chronic diseases. Therefore, there is a need of long-term clinical studies.

## 3. Cardiovascular Health

Cardiovascular diseases (CVDs) are a group of disorders of the heart and blood vessels that are the first cause of death globally. Unhealthy diet, physical inactivity, as well as tobacco and alcohol consumption are the main risk factors of CVDs [33].

Dietary fiber and other phytochemicals increase cardiovascular (CV) health parameters by affecting lipoprotein metabolism and inflammation response. Due to the antioxidant capacity and the anti-inflammatory activity, phytochemicals, especially phenolic compounds, can protect against atherosclerosis [34].

Puglisi et al. [35,36] reported that incorporating raisins into the diet or increasing walking steps have markedly beneficial effects on CV risk and satiety. In their study, 34 men and postmenopausal women were randomly assigned to three groups: (1) to consume 160 g raisins per day, (2) to increase the number of walking steps per day, and (3) a combination of interventions 1 and 2. After six weeks, results showed a decreased in total and LDL cholesterol (LDL-c) content in all subjects. In those who consumed raisins, the decrease in LDL-c was suggested to be caused by the up-regulation of the LDL receptor, as well as the higher intake of fiber and the polyphenol interference with cholesterol absorption [36]. In addition, systolic blood pressure and plasma soluble intercellular adhesion molecule-1 (sICAM-1) levels were reduced. Lower levels of sICAM-1 might prevent the evolution of atherosclerosis by diminishing monocyte adhesion to the vascular endothelium [35]. Raisin consumers also had lower levels of tumor necrosis factor α (TNF-α), a powerful pro-inflammatory cytokine, whose reduction could potentially prevent the progression of inflammatory damage.

Similar results were obtained in a more recent intervention involving 33 apparently healthy smokers with a low adherence to the Mediterranean diet [37]. For four weeks, all participants consumed less than five daily servings of fruits and vegetables and the intervention group consumed raisins equal to five fruit servings (90 g/day). At the end, ICAM-1 levels were significantly reduced in subjects over 30 years old in the intervention group. The women in this group had significantly lower diastolic blood pressure, total cholesterol and LDL-c compared to baseline. However, no differences were found in levels of inflammatory and oxidative stress markers. The explanation given for this was that the total antioxidants supplied by the daily consumption of raisins was not enough to neutralize ROS produced by smoking. Nevertheless, the duration of the study was short, and the sample size was small, so no realistic conclusions could be exported.

In the case of healthy but overweight individuals following a low-flavonoid diet, daily consumption of seedless raisins (90 g) for 14 days modestly increased serum antioxidant capacity [38]. However, there were no favorable changes in fasting or postprandial inflammatory markers, suggesting a lack of connection between biomarkers of total plasma antioxidant capacity and inflammatory status in this study population, or that the antioxidant capacity was not enough to alter inflammation. In fact, the authors believed that the reason why there was no alteration in the oxidative stress and inflammation was probably due to the insufficient quantity of raisins provided in the study, and it had nothing to do with the duration of the intervention. The dose of two servings/day of raisins that they supplied (90 g) contained around 68–94.5 mg/day of phenolic compounds.

### 3.1. Hypercholesterolemia

Hypercholesterolemia, a risk factor for CVDs, has become the most frequently encountered medical problem worldwide, bad dietary habits being among its principal causes [39].

An intervention study was carried out with 15 hypercholesterolemic adults to evaluate the hypolipemic effects of a plant-based diet rich in whole grains, nuts and sun-dried raisins on serum lipoproteins [40]. After four weeks of intervention, the total fat intake remained unchanged, although there was a shift in fatty acid distribution in plasma: monounsaturated and polyunsaturated fatty acids (PUFA) increased, whereas saturated fatty acids (SFA) diminished. An increase in triglyceride and fiber intake was accompanied by a reduction in dietary cholesterol, total cholesterol, LDL-c and HDL-c.

Later, the same authors designed a similar crossover study with 12 hyperlipidemic women [41]. It lasted 8 weeks. During the first four weeks, participants consumed refined and processed foods, and for the last four weeks they changed to a phytochemical-rich diet based on whole grains, legumes, fruits, vegetables, nuts and seeds (including three servings of 42 g/day of raisins). Changing from the refined-food diet to the phytochemical-rich diet caused a 61% reduction in SFA and an 81% increase in PUFA, as well as a significant increase in carbohydrate intake and a significant decrease in protein intake. At the end of the study, total cholesterol and LDL-c content were reduced by 13% and 16%, respectively, compared to the baselines.

However, the results of these two studies cannot be attributed exclusively to raisins, but to the diet followed based on whole and unrefined foods.

### 3.2. Hypertension

Borgi et al. [42] investigated the association of individual fruit and vegetable ingestion with the risk of developing hypertension in three large prospective cohort studies with 187,453 participants and more than 20 years of follow-up. Total whole fruit intake was correlated with a lower risk of developing hypertension, whereas total vegetable intake was not. Regarding raisins, the consumption of ≥ 4 servings/week (one serving = 40–50 g) was associated with a lower hypertension risk.

Blood pressure, a risk factor for hypertension, seems to be reduced after consuming 84 g of raisins per day [43,44]. According to the authors, the increased intake of dietary fiber and potassium contained in raisins might contribute to lowering blood pressure. In addition, polyphenols may play a role in promoting nitric oxide release and reducing blood pressure [45,46].

Raisin intake seems to lower total cholesterol, LDL-c, and some inflammatory biomarkers. However, there is lack of interventional studies focusing on the effects of eating raisins on CV health of healthy individuals and individuals with CV risk factors. More clinical trials should be performed to draw a firmer conclusion.

## 4. Diabetes

Glycemic index (GI) describes the blood glucose response after ingestion of a carbohydrate-containing test food relative to a carbohydrate-containing reference food, generally glucose or white bread [47]. The insulin index (II) has been defined as a direct index of the postprandial insulin response to a test food in comparison with an isoenergetic portion of a reference food (glucose or white bread) [48]. Foods are classified as having a low (< 55), medium (55–69) or high (> 70) GI or II [47]. Raisins have a low-to-moderate GI and II, making them a healthy choice for individuals with diabetes or insulin resistance. These indexes have been analyzed by several authors [49,50].

GI values for raisins in sedentary and prediabetic people were low (≤ 55), and moderate in aerobically trained individuals (55–69), whereas II was low for all groups (≤ 55) [50]. The GI value for raisins in healthy adults was 64 [51] and more recently a value of 49 was obtained [49]. Overall, reported GI values range from 49 to 69 and II values from 47 to 54. Fructose, which is the most abundant sugar in raisins, seems to be responsible for these index values, since it has a low GI of 15 ± 4 [52].

In addition to foods with a low GI and high fiber content, another important dietary factor in the management and prevention of diabetes is a high intake of antioxidants, especially phenolic compounds such as flavonoids. It has been hypothesized that flavonoids preserve cell function by lowering oxidative stress damage and protect against the development of insulin resistance to type 2 diabetes (T2D) [53,54]. Anthocyanins have also been studied for their antidiabetic properties, including the prevention of free radical production and lipid peroxidation, reduction of blood lipids and hemoglobin A_1c_ (HbA_1c_), increased insulin secretion, and improvement of insulin resistance. However, further large-scale and long-term clinical trials are required to draw a solid conclusion about the contribution of anthocyanins to the management and prevention of diabetes [55].

Impaired fasting glycemia (IFG), also known as pre-diabetes or metabolic syndrome, occurs when blood glucose levels in the body are increased during periods of fasting, but not enough to prompt a diagnosis of diabetes [56]. Byrne et al. [57] compared blood glucose and insulin responses to three pre-exercise snacks before, during and after exercise in men with IFG and men with normal fasting blood glucose values in a randomized and controlled crossover study. The snacks consisted of (1) 296 mL of water and 296 mL of a glucose-tolerance test beverage with a GI of 100, (2) 69 g of raisins with a GI of 49, and (3) 77.4 g of chocolate-flavored energy bar with a GI of 58. Results showed that serum glucose concentrations were higher in the IFG group, whereas no significant differences were found in serum free-fatty-acid concentration. Moreover, serum insulin concentrations took longer to return to baseline in IFG men because of the higher serum glucose concentration. Raisins reduced both the postprandial glycemic and insulinemic responses during exercise, which is helpful for those with IFG, as well as for those with normal fasting glucose. Therefore, consuming a low-GI snack before exercising seems to be favorable from the perspective of insulin response.

Health professionals in the USA, free of major chronic diseases at baseline, participated in a prospective longitudinal cohort study to determine whether individual fruits were differentially associated with a risk of T2D [58]. Although total whole fruit consumption was weakly related with a lower risk of T2D, the associations differed significantly among individual fruits. Greater intake of specific whole fruits, particularly grapes, blueberries and apples, was significantly correlated to a lower risk of T2D, whereas higher fruit juice intake was associated with a higher risk. In the case of grapes and raisins, consumption was linked to a 19% lower risk of T2D compared to drinking fruit juice 3 times a week.

Some interventions have been carried out with diabetic individuals to determine the effects of consuming raisins. In a 12-week randomized study, the effect of daily consumption of dark raisins versus alternative processed snacks on glucose levels and other cardiovascular risk factors was evaluated in 51 patients with T2D [43]. Dark, dry Californian raisins were given as 28 g servings (90 kcal) three times per day (before breakfast, lunch and dinner). Those participants who consumed raisins had a significant 23% reduction in postprandial glucose levels compared to the group receiving alternative processed snacks. A 19% reduction in fasting glucose was also observed, although it was not of statistical significance. Anderson et al. [44] performed a similar randomized trial, but in this case the participants were patients with prehypertension and mild hyperglycemia. In accordance with the previous study, raisins reduced postprandial glucose levels. Moreover, it was found that the HbA_1c_ level was reduced.

Another randomized crossover study was carried out with 15 healthy subjects and 15 diabetic patients [59]. They received 74 g of Corinthian raisins or 50 g of glucose as a reference food. Blood samples were collected before and at 30, 60, 90, 120, 150 and 180 min after raisin or glucose consumption. The peak of serum glucose was at 30 min in healthy subjects and at 60 min in T2D, for both treatments. Further, the peak of serum insulin was at 30 min in healthy and at 90 min in T2D, for both treatments. Results showed that raisin intake reduced glucose and insulin responses, both in healthy and diabetic subjects. The same crossover study was performed with 10 healthy normal-weight subjects, but no differences were found in glucose and insulin responses between raisin or glucose consumers [60].

Patients with T2D took part in a randomized, controlled, 24-week prospective intervention trial. All participants consumed fewer fruits and vegetables than the recommended amount of five servings daily, but the intervention group consumed Corinthian raisins equal to two fruit servings (36 g/day) whereas the control group did not consume grapes or raisins. Patients that consumed raisins had a significantly reduced diastolic blood pressure and higher total antioxidant potential compared to baseline [61]. Scientific evidence suggests that eating fruits with high amounts of flavonoids results in lowering blood pressure [62]. In fact, in an animal model study, quercetin present in Corinthian raisins was shown to play a key role in blood pressure regulation by activating calcium potassium channels in isolated myocytes from rat coronary arteries, which results in the relaxation of the coronary artery [63].

Raisins are a good choice both for diabetics and healthy individuals since they have low-to-moderate GI, thus their consumption lowers the glycemic and insulinemic responses. Moreover, they seem to reduce blood pressure in patients with T2D, which is a risk factor for CVD.

## 5. Intestinal and Colon Health

The effect of consuming raisins on intestinal transit time, fecal weight and fecal bile acids was evaluated by Spiller et al. [64]. A total of 16 healthy adults consumed 84, 126, and 168 g raisins per day, each for two weeks. Results showed that as raisin intake increased, so did the fecal weight, while the transit time was shorter. Moreover, fecal bile acids (a possible indicator of colon cancer risk) decreased when two servings of raisins were consumed per day (84 g). Therefore, two servings of raisins per day seem to induce beneficial changes in colon function.

The same authors conducted a crossover intervention with 13 healthy adults who ate 120 g/day of raisins or 5 g/day of tartar (equivalent to tartaric acid in 120 g of raisins) for 9 weeks [17]. As reported previously, raisin intake decreased intestinal transit time and fecal bile acids and increased fecal weight. Furthermore, raisins increased total short-chain fatty acid (SCFA) excretion. Tartaric acid produced the same results, although it did not affect fecal weight. The authors postulated that both the dietary fiber and tartaric acid mediate the intestinal beneficial effects of raisins, including a decrement of the risk of colon-rectal cancer.

Colon function was also improved after following a plant-based diet rich in whole grains, nuts and sun-dried raisins [40] or a phytochemical-rich diet based on whole grains, legumes, fruits, vegetables, nuts and seeds [41].

The microbiota has gained especial interest since research has demonstrated their key role in maintaining a good human health [65]. However, it is still a novel field and few studies have been published concerning food and microbiota. In the case of raisins, only two studies have been performed: one in vitro and one in vivo.

The effect of raisins on the composition of the human gut microbiota was assessed *in vitro* by Mandalari et al. [66]. To do this, they used a full dynamic gastric and colon model to simulate the full gastrointestinal tract (mouth, stomach, small intestine and large bowel). The colonic fermentation was done with fecal material supplied by a healthy donor. The intake of fructooligosaccharides (FOS) or raisins increased total anaerobes and Bifidobacterial and Lactobacilli after 8 and 24 h of incubation, and Clostridia slightly increased after 4, 8, and 24 h of incubation only with raisins. The Bacteroidetes decreased after 4 h of incubation only in the presence of raisins. Regarding the taxonomic composition of the microbiota, when raisins were supplemented there was a loss in 13 species after 24 h of fermentation, whereas the supplementation with FOS increased the diversity by 91 species. Firmicutes decreased with raisin supplementation, whereas they increased with FOS. An increase in Proteobacteria and Actinobacteria was observed with both supplements. The numbers of *Faecalibacterium prausnitzii* and Ruminococcaceae decreased with raisin supplement, whereas *Roseburia* spp. increased. Total SCFA production increased with FOS and raisin supplementation. These findings demonstrated that raisins have a great potential to promote colonization and proliferation of beneficial bacteria, such as Bifidobacterial and Lactobacilli, in human microbiota and stimulate the production of SCFA. This could be attributed not only to the FOS content of raisins, but also to the phenolic compounds and tartaric acid. However, according to the results obtained, it may seem that raisins do not contribute to the biodiversity of the microbiota, whereas FOS by themselves do.

In a recent in vivo study on how raisins could affect gut microbiota composition, healthy individuals consumed three servings (28.3 g each) of sun-dried raisins daily for 14 days. Adding raisins to the diet did not significantly affect overall microbiota diversity but altered the prevalence of specific operational taxonomic units (OTUs), which are used to categorize bacteria based on sequence similarity [67]. Moreover, an increased production of SCFA was found in fecal samples. The levels of OTUs matching *Faecalibacterium prausnitzii*, *Bacteroidetes* sp. and *Ruminococcus* sp. increased compared to baseline, while those closest to *Klebsiella* sp., *Prevotella* sp. and *Bifidobacterium* spp. decreased [68]. Higher levels of *F. prausnitzii* have been associated with reduced chronic inflammation [69]. *Ruminococcus* sp. and *Bacteroidetes* spp. produce SCFA, which contribute to maintaining a balanced gut ecosystem. The significant reduction in OTUs matching *Klebsiella sp.* may suggest a reduced risk of enteric inflammation or urinary tract infections [68]. The number of OTUs that significantly changed compared to baseline was greater during week 1 than week 2, probably because introducing raisins into the diet has mostly short-term effects on the microbiota. However, the study was limited by the small number of participants, so these results are not representative and further research needs to be done.

No firm conclusions can be drawn concerning raisins and microbiota, since there is a lack of scientific evidence. More research should be done, especially in vivo, to assess the effects of consuming raisins on gut microbiota.

## 6. Dental Health

The fact that raisins adhere to the teeth and contain high amounts of sugar have misled people about their promotion of dental caries [70]. Nonetheless, recent reports refute these traditional thoughts.

Three conditions are thought to promote the development of dental caries: (1) low oral pH, (2) adherence of food to teeth and (3) biofilm or bacterial behavior. When raisins are consumed alone, they (1) do not reduce the oral pH below the threshold of 5.5, which would favor enamel demineralization, (2) do not remain on the teeth for too long and (3) contain antioxidants with antibacterial activity [70]. Raisins may also benefit oral health because they possess antimicrobial phytochemicals that inhibit the growth of oral bacteria associated with dental diseases.

Utreja et al. [71] conducted a randomized control study with 20 healthy children between seven and 11 years old to investigate the effects of four foods on in vivo plaque acidogenicity. These foods were raisins, bran flakes, commercial raisin bran cereal and experimental raisin bran cereal with no added sugar (eRB). Sucrose and sorbitol (10%) were used as positive and negative controls, respectively. After the intake of raisins and eRB, the dental plaque pH never fell below 6.0 and thus never reached the 5.5 threshold. They suggested that raisins do not reduce oral pH because they contain low concentrations of sucrose, which is the main substrate for the bacteria that contribute to dental plaque formation. Instead, raisins are rich in glucose and fructose. Moreover, the researchers observed that raisins rapidly cleared from the mouth and were not very retentive on the tooth surface.

In 2008, Rivero-Cruz et al. [16] investigated the antimicrobial activity of compounds isolated from raisins and some derivatives against two oral pathogens: *Streptococcus mutans* and *Porphyromonas gingivalis*, linked to caries and periodontal disease, respectively. The compounds oleanolic acid, oleanolic aldehyde, 5-(hydroxymethyl)-2-furfural, and the derivative oleanolic acid sodium salt inhibited the growth of the test bacteria at concentrations ranging from 3.9 to 500 µg/mL. Catechins have also shown to exert a bactericidal effect against *S. mutans* and *Streptococcus sobrinus*, to prevent adherence of bacteria to teeth and to inhibit two enzymes, glucosyl transferase and amylase, that could increase dental caries [72].

## 7. Diet Quality

Diet is one of the key factors that contributes to keep a good health. Although diet quality is a heterogeneous and multidisciplinary term, different indexes have been proposed to assess it. Scientific reports have demonstrated a correlation between eating patterns and healthy growth and development throughout childhood and adolescence, as well as, the prevention and/or mitigation of health problems in adults [73]. The Mediterranean diet is a clear example [74]. It focuses on the daily intake of whole grains, olive oil, fruits, vegetables, legumes, nuts, herbs, and spices. Thus, knowing and understanding food composition and its impact on human health is crucial to establish dietary guidelines and predict the quality of a diet.

In the case of raisins, only two reports carried out in children were found to evaluate the diet quality when consuming these dried fruits. The first one, in 2012, showed that the consumption of a premeal snack of raisins reduced meal-time energy intake and did not lead to increased cumulative energy intake in children 8–11 years old [75]. These children randomly received ad libitum snacks (raisins, grapes, a mix of almonds and raisins, or water) or 150 kcal fixed treatments. Both treatments were followed by an ad libitum pizza meal 30 min later. Both the ad libitum and fixed-calorie raisin snack reduced the intake of pizza (and therefore food) compared with the water control, also reducing the appetite.

Later, Fulgoni et al. [76] examined the association between the ingestion of raisins and raisin-containing foods with nutrient intake and dietary quality in children and adolescents 2–18 years of age. The study found that raisin consumers had significantly higher daily intakes of dietary fiber (22.9%), potassium (16%) and magnesium (11.6%), and lower intakes of added sugar (−19.1%), monounsaturated fat (−9.2%) and total fat (−5.1%) compared to non-consumers. Consumers of raisin-containing foods also had similar results. In addition, total fruit, whole fruit and whole grain intake was significantly higher for both groups compared to non-consumers. In conclusion, overall diet quality was better among consumers of raisins or raisin-containing foods.

The studies found in adults evaluated appetite hormones and satiety. Glucagon-like peptide-1 (GLP-1) and gastric inhibitory peptide (GIP) are gastrointestinal peptide hormones, also known as incretins, that trigger insulin release after the ingestion of glucose in healthy individuals [77]. Ghrelin is an appetite-stimulating gastrointestinal hormone [78]. One study observed significant differences for ghrelin and GIP, whose levels were lower after raisin compared to glucose consumption [60]. The second study found that plasma leptin and ghrelin concentrations increased in raisin consumers, as well as in individuals who walked more steps per day [36]. Feelings of satiety also increased when a plant-based diet rich in whole grains, nuts and sun-dried raisins was followed [40].

Although there is a small number of studies to draw a clear conclusion, these results suggest raisins as a healthy snack. Despite being a high-energy food and containing more than 50% of sugar, they seem to reduce food intake and appetite, probably due to their fiber content, and they bring nutrients that are necessary for the human body functionality and metabolism. In addition, appetite hormones seem to increase, which may be also involved in the loss of appetite. However, more research should be done concerning the impact of eating raisins on cumulative energy and body weight both in children and in adults.

## 8. Cell Line and Animal Models

Despite being criticized, cell line and animal model studies represent a first approximation to predict and understand the effects in humans. Intervention and clinical trials must be performed to confirm solid conclusions. However, cell line and animal model studies can reveal interesting findings that can lead to perform in vivo studies with humans.

### 8.1. Cell Line Models

Some researchers have proven the antioxidant activity of the polyphenol extract of raisins in different cell lines. In peripheral blood mononuclear cells from healthy volunteers the polyphenol extract of raisins inhibited LDL oxidation and increased the levels of antioxidant glutathione (GSH). The DPPH radical scavenging assay also showed that the extracts had good antioxidant potential. These results were correlated to the polyphenol content. Besides, they also found a correlation between total polyphenol content and cell survival when a stressor agent (*tert*-butylhydroperoxide (tBHP)) was applied [25].

Di Lorenzo et al. [79] studied the anti-inflammatory activity of 5 raisin hydro-alcoholic extracts on IL-8 at gastric level. IL-8 is a cytokine that plays a key role in gastric diseases and it is released by gastric epithelial cells during gastric inflammation. The study was conducted with human adenocarcinoma cells (AGS). Only the Turkish variety significantly inhibited IL-8. Catechins and procyanidins, together with other unknown components, seem to contribute to the anti-inflammatory activity of the extracts.

Two reports were found focusing on the chemopreventive properties of raisins, both carried out with cell lines.

The first one investigated the gastric preventive activity of methanol polyphenol extracts from currants and sultanas to inhibit cell proliferation, induct apoptosis and inhibit inflammation in AGS cells derived from fragments of a gastric tumor. Different volumes of the extracts (corresponding to 500, 400, 250, and 100 µg of dried product of the methanol extracts) were added in 10^5^ cells for 24 h. AGS cells were preincubated with these doses of the extracts and stimulated with cytokine TNF-α to assess the inflammation response. On the one hand, all extracts from 500 µg suppressed cell proliferation through triggering apoptosis, and on the other hand, they reduced protein and mRNA levels of ICAM-1 when cells were stimulated by TNF-α. They found a statistically significant negative correlation between ICAM-1 levels and polyphenol concentration, which might suggest the role of this compounds in the anti-inflammatory and antiproliferative responses. AGS growth was mostly inhibited by the extracts from 500 µg of two varieties (Cretan sultana and Nemea currant). Nevertheless, there was not any significant reduction in IL-8 levels. The fact that there were no changes in IL-8 levels, whereas ICAM-1 decreased, suggested the involvement of the NF-κB regulation but not the AP-1 transcription factor. This study proved for the first time the chemopreventive activity of currant and sultana phenolic extracts by limiting the proliferation of human gastric carcinoma cell through triggering apoptosis and suppressing ICAM-1 levels. However, the mechanisms by which the apoptosis is induced and the specific bioactive compounds responsible of it are not clear and further investigations should be done [80].

The second study investigated the effects of methanol phenolic extracts of Corinthian raisins and sultanas on human colon cancer cells (HT29). Different concentrations of the extracts, corresponding to 500, 400, and 250 µg of dried product, were added in cells, together with the stimulator agent TNF-α. IL-8, NF-κB p65 (a subunit of NF-κB) and cyclooxygenase-2 (COX-2) were evaluated. Both raisin varieties suppressed cell proliferation at doses of 500 and 400 µg. Further, a decrease in the levels of glutathione, COX-2, IL-8, and NF-κB p65 were achieved at the dose of 500 µg. For the first time, the chemopreventive properties in colon cancer cells were demonstrated, proving that the phenolic extract of Corinthian and sultana raisins prevent cell proliferation and inflammatory response, being sultanas the ones with a higher activity. The DPPH radical scavenging assay also revealed the great antioxidant capacity of the extracts. The dose of 500 µg of extract corresponds to approximately 1–2 g of raisins [81]. Like the previous study, the mechanisms by which these effects happen are still unclear and further research must be done to fully understand it.

### 8.2. Animal Models

Several studies have also been conducted with animal models. The oldest dated from 2010 and aimed to determine the effect on restoring bone loss by supplying certain functional foods to a rat model of osteoporosis. The diets involved (1) whole food (dried plum (DP), figs, dates, raisin or blueberry), (2) fractionated food (DP puree, DP juice or DP pulp/skin), or (3) isolated components (DP polyphenols, FOS or β-hydroxy-β-methylbutyrate). Results showed that DP improved the right femur and fourth lumbar bone mineral density of rats, and even better results were found when FOS were supplemented. In the case of raisins, it had a positive effect at the fourth lumbar [82]. Raisins are one of the top contributors of boron in the diet, a mineral involved in the skeletal maintenance [83]. The findings of this study suggest the possibility of a site-specific action of the bioactive components, thus additional investigation is needed to a better understanding.

To investigate the antioxidant effects of raisin extracts “Karkni” on diabetes, alloxan-induced diabetic rats were used [84]. Alloxan, a glucose analogue, is a toxin that damages the pancreatic *β* cells, thus severely diminishing the levels of insulin [85]. Concentrations of 125, 250 and 375 mg/kg of raisin extract were supplied to rats for four weeks. The dose of 250 mg/kg resulted to be the most effective in reducing blood glucose levels, although the three treatments demonstrated a hypoglycemic effect. This effect was associated with the carbohydrate and flavonoid content of raisins. Plasma triglycerides, total cholesterol and LDL-c decreased after the administration of the three doses. The high levels of HbA_1c_ in diabetic rats were restored to its normal values after being treated with the raisin extracts. Likewise, uric acid levels decreased after the treatment, which is favorable since this molecule is a predictor of increased cardiac complications [86]. This research revealed the potential role of raisins in preventing or reducing the complications of diabetes thanks to its hypoglycemia and hypolipidemia effects. However, clinical trials with diabetic patients should be performed to corroborate these beneficial effects.

The effect of consuming raisins on rats with hypercholesterolemic kidney was also examined [87]. Animals were divided into four groups and for 13 weeks they received: (1) a standard diet along with saline (control group), (2) a standard diet along with 0.5 g of raisins, (3) a high-cholesterol diet (HCD), and (4) a HCD along with 0.5 g of raisins. At the end of the study, kidneys were evaluated. Rats that followed the HCD experimented an increase in blood glucose and insulin levels as well as in the cholesterol, triglycerides and LDL-c levels, and a decrease in HDL-c levels. The addition of raisins to the HCD resulted in a significant decrease of these values, except from the HDL-c, whose levels increased. The hypolipidemic effect of raisins may be attributed to is phenolic and fiber content, which could probably difficult the cholesterol absorption [88]. Furthermore, the administration of raisins ameliorated the histopathological damages of the kidney occasioned by the HCD, which demonstrated the potential of this dried fruit to protect the kidney from a hypercholesterolemia-induced injury. The hypoglycemic activity of raisins was suggested to be attributed to their flavonoid constituents as Ghrairi et al. observed [84].

Recently, a similar study has been published. In this case, red grape juice (RGJ) and dark raisins (DR) were assessed for protecting the renal tubules against HCD. Rats were divided into four groups and for 12 weeks they received: (1) a standard diet (control group), (2) a HCD, (3) a HCD along with RGJ, and (4) a HCD along with DR. Feeding rats with HCD altered the structure of renal tubules. However, the administration of both RGJ and DR significantly improved the lipid profile, the HDL-c, reduced the blood glucose, increased the insulin level and ameliorated the histopathological alteration in the renal tubules [39]. Again, the renoprotective effect of raisins was demonstrated. The findings of these two studies may indicate the benefits of consuming raisins especially for those who followed a high-fat diet. However, human assays must be performed to draw serious conclusions.

The last study found with animal models has been published recently and it is the first paper concerning the role of raisins in Alzheimer’s disease (AD) [89]. Little is known about this disease and great efforts are being made to investigate how to prevent and attenuate it. The rats of this study were divided into four groups: (1) AlCl_3_ + 6 g/day of raisins, (2) AlCl_3_, (3) 6 g/day of raisins, and (4) control group. AlCl_3_ induces AD. After 60 days, the rats that were treated with AlCl_3_ showed degenerated neurons and abnormal histological architectures. The addition of raisins attenuated these negative effects. In fact, it was demonstrated that treatment with raisins decreased the time taken for rats to find a hidden platform, thus proving their efficiency in reducing the harmful effect of Al on learning and memory. Phenolic compounds, as well as other antioxidant components, might be important characters involved in these beneficial effects. These favorable results open the door to new studies evaluating the potential effects of raisins on preventing and attenuating the symptoms of neurodegenerative diseases. It would be interesting to conduct human intervention assays with pre-AD or AD patients and see if there is clinical evidence to support the benefits of raisins.

## 9. Conclusions

According to the scientific evidence presented in this review, despite their high content of sugar, raisins are a source of beneficial components and a healthy snack. Due to their composition, they contribute to a better diet quality, and their consumption before a meal could be favorable for regulating appetite in normal-weight healthy subjects. Eating raisins may reduce hunger and affect dietary intake by altering hormones influencing satiety, thus diminishing the energy intake of the meal, which in turn could help to maintain a correct body weight. Their antioxidant capacity has been extensively demonstrated and correlated to the phenolic content, and although this may be an indication of their potential to exert beneficial effects on human health, more scientific evidence in intervention studies is required. Due to their phenolic components and high fiber content, raisins may improve cardiovascular health parameters by increasing the plasma antioxidant capacity and lowering total and LDL cholesterol levels, systolic blood pressure and molecules linked to inflammation response. Incorporating raisins to the daily diet seems to lower some CV risk factors, even though these effects were not appreciated in overweight individuals. Moreover, raisins have a low-to-moderate GI, which makes them a healthy choice for diabetics or those with insulin resistance, and their consumption could be linked to a lower risk of T2D. The potential of raisins to preserve a good dental health has also been demonstrated, due to their antibacterial activity, low adherence to teeth and an oral pH not below the threshold that damages enamel. Raisin intake might also be favorable for colon function and their prebiotic content seems to affect gut microbiota. It would be of great interest to perform more studies concerning the impact of raisin intake on gut microbiota and colon function before drawing any clear conclusion. Cell line and animal model studies have shown interesting results, suggesting the investigation of consuming raisins in other diseases, such as cancer and Alzheimer’s disease, but in intervention studies with humans. Although raisins have shown to be a potential beneficial food, deeper and further research is required to state whether eating raisins could be favorable and beneficial for preserving a good health. Overall, with the research done so far, it seems that adding 80–90 g of raisins to the daily diet may be favorable for human health. However, more intervention studies with specific biomarkers are required.

## Figures and Tables

**Table 1:** Nutritional composition of golden and dark seedless raisins (100 g) [8].

Nutrient	Golden Raisins	Dark Raisins	Units
**Proximates**			
Water	14.90	15.46	g
Energy	301	299	kcal
Protein	3.28	3.30	g
Total lipid	0.20	0.25	g
Carbohydrate (by difference)	80.02	79.32	g
Fiber (total dietary)	3.30	4.50	g
Sugars (total)	65.70	65.18	g
**Minerals**			
Calcium	64	62	mg
Iron	0.98	1.79	mg
Magnesium	35	36	mg
Phosphorus	101	98	mg
Potassium	746	744	mg
Sodium	24	26	mg
Zinc	0.37	0.36	mg
**Vitamins**			
Vitamin C (total ascorbic acid)	3.20	2.30	mg
Thiamin	0.008	0.106	mg
Riboflavin	0.191	0.125	mg
Niacin	1.142	0.766	mg
Vitamin B-6	0.323	0.174	mg
Folate (DFE) ^1^	3	5	µg
Vitamin B-12	0	0	µg
Vitamin A (RAE) ^2^	0	0	µg
Vitamin A (IU) ^3^	0	0	µg
Vitamin E (alpha-tocopherol)	0.12	0.12	mg
Vitamin D (D2 + D3)	0	0	µg
Vitamin D	0	0	IU
Vitamin K (phylloquinone)	3.5	3.5	µg
**Lipids**			
Fatty acids (total saturated)	0.065	0.094	g
Fatty acids (total monounsaturated)	0.014	0.024	g
Fatty acids (total polyunsaturated)	0.057	0.053	g
Fatty acids (total *trans*)	0	0.001	g
Cholesterol	0	0	mg

^1^ DFE (dietary folate equivalents); ^2^ RAE (retinol activity equivalents); ^3^ (IU) International Unit.
